# From trust to skepticism: An in-depth analysis across age groups of adults with sickle cell disease on their perspectives regarding hydroxyurea

**DOI:** 10.1371/journal.pone.0199375

**Published:** 2018-06-27

**Authors:** Cynthia B. Sinha, Nitya Bakshi, Diana Ross, Lakshmanan Krishnamurti

**Affiliations:** 1 Department of Pediatric Hematology-Oncology-BMT, Emory University, Atlanta, Georgia, United States of America; 2 Aflac Cancer and Blood Disorders, Children’s healthcare of Atlanta, Atlanta, Georgia, United States of America; 3 Department of Pediatric Hematology-Oncology, Children’s Hospital of Pittsburgh, Pittsburgh, Pennsylvania, United States of America; 4 University of Pittsburgh, Pittsburgh, Pennsylvania, United States of America; University of Texas at Austin Dell Medical School, UNITED STATES

## Abstract

Despite its efficacy, the uptake of HU in adults with sickle cell disease (SCD) is poor likely due to a combination of system, provider, and patient-related factors. We investigated attitudes of adult patients towards HU by conducting qualitative interviews with 95 adult SCD patients (age 18 to 67 years old, 71 were female). While 53% of all participants reported that they were currently taking HU, patients ranging in age 18–30 years (Group 1) were more likely to report current HU use as compared to those (Group 2) ranging in age 31–67 years (65% vs. 41% P = 0.01). Most Group 1 participants who reported currently taking HU indicated that the decision to start HU was made by a parent, though some made the decision themselves as a young adult. Group 1 participants expressed trust in the efficacy of HU as well as trust that their physician adequately shared risks and benefits for the medication. The Group 2 participants, who were not currently on HU, were skeptical that all the risks and benefits of HU were known, were concerned that the efficacy of HU was not proven, and that they were not receiving complete information about its potential side effects. Of Group 2 participants who reported currently being on HU, 25% were concerned about the side effects and efficacy of HU and reported continuing HU because of a lack of effective alternatives. These data suggest that there are significant differences by age in adult SCD patients’ attitudes towards, utilization and understanding of the risks and benefits of HU.

## 1. Introduction

Sickle cell disease (SCD) is associated with substantial morbidity, quality of life, healthcare utilization, and premature mortality. Hydroxyurea (HU) is the first disease modifying therapy approved by the FDA for adults with SCD. HU has been demonstrated, in both adults[1] and children[2], to reduce morbidity and to decrease mortality in adults with SCD [3]. Despite demonstrated efficacy in clinical trials, HU remains underutilized [4] [5] [6, 7], due to a combination of health-care provider, system, and patient-level factors. Lack of knowledge about HU, doubts regarding effectiveness, concerns over side effects and unrealistic expectations from HU treatment have previously been identified as barriers to the wider uptake of HU by patients [8]. Since approval of HU for SCD by the FDA in 1998, there has been a progressive increase of the use of HU in SCD. It was originally reserved for adults with sickle cell anemia (SCA) who had severe manifestations of disease as described in the Multicenter Study of HU trial[1]. Following the demonstrated efficacy in the BABY-HUG study [2] the current recommendation is to offer it to all children nine months of age and older with HbSS and HbS-beta 0 thalassemia regardless of disease severity [9]. As evidence regarding the benefits of HU has dramatically increased during their lifetime, adult patients with SCD are likely to have received changing and sometimes conflicting treatment recommendations regarding HU.

Prior research has identified barriers to initial uptake and adherence to HU[8, 10]. In general, adult patients lacked general knowledge of HU and were overly concerned about side effects[8]. For adult patients who stopped HU, many doubted the efficacy stating they continued to have pain crises despite being on HU[8]. When introduced with comprehensive information, parents of pediatric patients are very likely to start HU, though parents were also concerned about side effects and efficacy[10]. There is, however, a paucity of information on how attitudes and understanding of HU may vary by age of the patient. Since the approach to the use of HU has evolved over time, it is likely this may have impacted how patients of different ages view HU and its place in treatment of SCD. Furthermore, there is a lack of in-depth qualitative studies that examine adult patient’s attitude towards disease modifying therapies, such as HU in SCD. We focused our initial analyses on attitudes towards HU as a model for how SCD patients viewed their disease and treatment options. After this initial stage of analysis, a pattern emerged that indicated there may be age-related differences in uptake and understanding of HU. Thus, we asked the question, “what are the attitudes amongst our sample population that represent barriers to uptake and utilization of HU and do these attitudes differ by age groups?”

## 2. Methods

### 2.1 Data collection

We conducted qualitative interviews with adult SCD patients from a geographically diverse population recruited from national conferences and two SCD clinics. Data were gathered as part of a larger study of decision making regarding disease-modifying therapies for SCD and development of a decision-aid tool for patients involving these disease-modifying therapies. For the larger study, participants were required to be 11 years or older at time of interview and have been diagnosed with SCD. No one was excluded from the study. The analysis for this manuscript only utilized the transcripts of participants 18 years and older. Recruitment and interviews were conducted by 2 men and 5 women; 4 of whom were physicians, one registered nurse, and two research assistants. As an incentive, participants were given a $25 gift certificate after completing the interview.

We used a semi-structured open-ended interview guide to collect data. The study was approved by the Institutional Review Board. Informed consent was obtained from all participants. Interviews were conducted either on the phone or in person. Audio recordings were transcribed verbatim. Transcripts were coded using qualitative content analysis with NVivo 11.

### 2.2 Data analysis

Transcribed interviews were analyzed using a line-by-line open coding as defined by Strauss and Corbin[11]. Coding did not begin as “tabula rasa,” but rather after an intensive literature review for attitudes towards treatment options for sickle cell patients and other chronic diseases [12]. Coding was derived from the participant responses. Once coding was completed, categories were developed from the patterns in the coding scheme with prevalence determined by simple content analysis. Thematic analysis was used to first illustrate the relationship between categories and then to address how the emergent theme addressed the research question. Manifest analysis coded specially for participants’ responses and comments concerning HU, the most common and least invasive disease modifying therapy. Once this phase of coding was completed, content analysis revealed the pattern of age and attitude towards HU. The participant responses to HU were coded as either negative or positive. For example, expressing satisfaction with efficacy was coded as positive whereas expressing concerned over unknown side effects was coded as negative. The positive and negative responses were eventually arranged under the larger categories of trust or skepticism. Content analysis determined the demarcation for grouping participants by age. The latent analysis was also developed from this phase of coding [13]. Participants’ overall attitude toward their chronic disease status as well as their healthcare provider was developed from this second round of coding.

We used a thematic approach to interpreting the coding scheme, though analysis ran concurrently with much of the coding. A theme represents something important to the research question and builds on existing literature about the overall topic. Additionally, a theme represents some level of patterned response or meaning within the data set [14]. The central theme, for this data set, was developed across age groups and then focused on the participants’ relationship to taking or not taking HU. Thematic saturation was achieved when we ceased to identify any new concepts related to our research question.

As a theme developed, the team of investigators discussed and substantiated each with examples provided. The lead coder has a PhD in sociology with an expertise in qualitative methodology and was responsible for developing the initial coding scheme. The coding scheme was demonstrated to the second coder, a registered nurse, who also has expertise in qualitative methodology. The second coder reviewed a large sample of transcripts to ensure inter-coder reliability. All of the authors reviewed analysis. Data analysis did not move forward until the team reached complete consensus.

## 3. Findings

### 3.1 Demographic data

Ninety-five patients with SCD participated in the interviews. Median age of the sample population was 32, (18–67) years. Seventy-one or 75% were female. Of the participants, 93 self-identified as African American, one as “other,” and one as “multiracial.” Approximately one-half of all participants self-reported that they were currently taking HU. HU use varied by age groups (Group 1: Age 18–30 and Group 2: Age 31–67). Demographic data were shown in Table 1 and HU utilization shown in Table 2.

**Table 1:** Demographics.

	Total = 95
**Age (median)**	32(18–67)
Female sex	71 (75%)
**Education Level**	
High school or GED	17(18%)
Some college	34(36%)
Bachelor degree	18(19%)
Terminal Degree (Masters, PhD)	18(19%)
Other	8(8%)
**Employment Status**	
Full time employment	18(19%)
Part-time employment	28(29%)
No employment	49(52%)
**Marital Status**	
Single, never married	66(69%)
Married	19(20%)
Divorced	9(9%)
Widowed	1(1%)

**Table 2:** HU utilization by age.

	HU Ever Prescribed		HU Ever Tried		Currently Taking HU	
Status	18 to 30 years old	31 to 67 years old	18 to 30 years old	31 to 67 years old	18 to 30 years old	31 to 67 years old
Yes	89%	71%	87%	74%	65%	41%
No	3%	27%	5%	17%	35%	59%
Unknown	7%	2%	8%	9%	0	0
Totals	n = 46	n = 49	n = 39	n = 35	n = 46	n = 49

Notes: Chi-square test (Yes/No) for HU Ever Prescribed and Currently Taking. Fisher exact test (Yes/No for HU Ever Tried.

HU Ever Prescribed (p = .030)

HU Ever Tried (p = .13)

Currently Taking HU (p = .017)

### 3.2 Emergent theme

The emergent theme was the influence of age of the participant on usage and understanding of HU. Content analysis revealed that the younger the participant, the more likely trust was expressed whereas around 31 years and older participants were more likely to express skepticism about the safety and efficacy of HU. This finding led us to generate the research question that there may exist age related differences in utilization and understanding of HU. The findings were organized by the theme of age-related differences in attitudes and understanding of HU. Group 1 were SCD adults aged 18 to 30 years and Group 2 were SCD adults 31 to 67 years. By each group, we discuss attitudes of participants currently on HU, those not currently taking HU, and lastly we analyzed the kind of information the participants reported they wanted to learn. This last finding may provide further insight into the age-related differences in utilization of HU.

### 3.3 Group 1: 18 to 30 years old

The majority of the Group 1 participants (65%) were currently taking HU at the time of the interview. Whether taking HU or not, most of the participants in this group had at some point been prescribed HU. Some of the participants were minors when their parent(s) or guardian made the decision to start HU, but some of the participants reported that they made the initial decision to start HU as young adults. For the participants currently on HU, none expressed dissatisfaction with the side effects or efficacy. Two participants discontinued HU and then later, as young adults, decided to go back on HU. Four of the 16 (35%) participants currently not taking HU had previously tried the drug but had stopped taking HU as adults. Providing further nuance to attitudes towards HU, we examined the topics concerning their health that the Group 1 participants reported they have discussed with their physician or intend to discuss.

#### 3.3.1 Group1: Currently on HU

Group 1 participants were able to articulate the mechanism of action for HU as well as see the benefits. Most of the participants mentioned that HU is a chemotherapy drug and described HU as “improving blood counts.”

“*Hydroxyurea is a form of a pill. It’s a type of chemo. What it does is it increases your fetal hemoglobin and causes your bone marrow to make more normal red blood cells, instead of sickle cells. And with it increasing the more normal blood cells which would last 120 days, it tells my body that I do not need to create any other type of sickled cells. It decreases the amount of sickling that I would have in my body*”

*(303, Female, 23yrs)*.

Group 1 participants were aware that the benefit from consistently taking HU resulted in the prevention of SCD complications. They were also aware that HU did not have an immediate effect on their health and that the benefits of consistently taking HU would occur over months or years.

“*Pretty much they said it was like a long term process kind of thing, because you’re not to actually start feeling any effects, it was supposed to take like, well they told me two months. They told me it was supposed to slow down the process of which sickle cell does it thing. Your organs and stuff break down a little bit faster, just a little bit. And they say the hydroxyurea is supposed to slow that down*.”

*(343, Male, 23yrs)*.

Group 1 participants currently taking HU rarely reported side effects. They provided details of the positive impact of HU on their lives. They shared, for instance, that they experience less pain, fewer crises, and consequently fewer hospital stays. These participants were also aware of indirect benefits of reducing opioid use while taking HU. That is, consistently taking HU may result in fewer pain crises, less need for pain medication, and a decrease in the loss of activity from work and school.

Participants who made the decision to start HU as an adult, trusted that HU was a good treatment choice and expressed how they felt about the decision to start HU. Overall, Group 1 participants expressed a trust in the potential for the positive outcome of taking HU.

“*I just thought of the pros and cons and I realized that I don’t have that many crises and it would be nice to like probably eliminate as much as I can. Because I like to, you know, work and walk and stuff. It all sounded like a good idea like what else can I lose? So that’s why I made the decision*”

*(377, Female, 19yrs)*.

Participants indicated that they would very much prefer to have less pain and fewer interruptions of school, work and social interactions. Group 1 participants currently on HU tended to also characterize positive interaction with their physician. They expressed a trust that the physician had adequately informed them about risks and benefits of taking HU.

#### 3.3.2. Group 1: Participants who were currently not taking HU

Of the 16 not taking HU, approximately half (n = 7) of Group 1 participants reported that it was never prescribed or it is unknown if a physician ever recommended the drug. For the remaining half (n = 9) who reported they were prescribed HU, four participants reported that they had tried the treatment. Aversion to taking HU stemmed from bad reactions, concerns about side effects, and in one case, perceived lack of efficacy. Perceived increase in crises was considered a bad reaction.

“*I was prescribed when I was 17, but I took it about 3 years and then I stopped due to I didn’t think it was helping me. I didn’t see a difference in pain crises and how frequent the crisis were, so I kind of discontinued that on my own*”

*(302, Male, 28yrs)*.

For these participants, they rarely discussed concerns that HU may be linked to infertility. It is unknown if the participants in Group 1 were concerned about fertility, though some participants were concerned about how SCD impacts family planning. Whether participants had actually tried HU or not, in general, they believed they were adequately informed about the risks and benefits of taking HU. Deciding not to take HU appeared to be most influenced on their belief concerning the need to manage their SCD.

For the seven participants who reported HU was never prescribed, some had heard of HU, but at least four appeared to be unfamiliar with the medication. For a few participants who reported HU was never prescribed, they had learned about the drug from other adults currently taking HU. These participants did not report that they discussed HU with their physician.

#### 3.3.3. Group 1: Information content- conversations with the physician

In addition to analyzing attitudes and experiences with HU, the interview guide questioned all participants on the types of information they would like to learn about SCD. The participants in Group 1 mostly requested more information on the pathophysiology of SCD. Specifically, participants wanted to understand the “genetics” of SCD. They wanted to understand how to interpret their lab results rather than receive a “good” or “bad” response from the healthcare provider. While some participants wanted to keep apprised of curative treatments, most were focused on understanding how to manage SCD complications in their daily activities though very few of the participants aged 18 to 30 reported any severe complications from SCD. Although not related to taking HU, a few participants wanted information family planning and parenting with SCD.

### 3.4. Group 2: 31 to 67 years old

Twenty-three of the forty-nine of the Group 2 participants (41%) were currently taking HU. The comparison to Group 1 currently taking HU is statistically significant (p .01). Thirty-five patients or 71% reported that HU had been recommended to them. Six patients had HU prescribed at some point, tried the medication and decided to stop the treatment. Fourteen of the participants in Group 2 who were not currently taking HU reported the treatment option had never been recommended. Consequently, they have never tried HU.

#### 3.4.1. Group 2: Currently on HU

Seventy-five percent of the participants in Group 2 currently taking HU reported little dissatisfaction with the efficacy of HU or the side effects.

“*And then my doctor talked about hydroxyurea. Well let’s try that and so that is how I got on the road to doing that. Until really the past 10 years I have not had any problems with pain from a sickle cell crisis at all. So I’ve been a happy camper in that regard*”

*(20235, Female, 65 yrs)*.

While these participants were satisfied with the results of taking the medication, some of these participants were not satisfied with the decision making process. They were dissatisfied with the lack of information the physician shared. These participants believed the healthcare provider (HCP) was too insistent about taking HU without providing a comprehensive treatment plan.

“*At the time I didn’t want to try it* [HU] *but then*, *I got to the point where my pain was just so bad that it was complicating my life*. *I was like why not give this a try*? *I don’t feel like it was really an in-depth conversation about*, *you know ‘This is how and why this is going to help*.*’ ‘This is why you should be on it*.*’ You know*, *I don’t feel like it was a real plan*”

(20257, Female, 37yrs)

Twenty-five percent of the Group 2 participants on HU expressed dissatisfaction with the drug and bemoaned the lack of available treatment options in general. Although they were dissatisfied with the side effects, they believe they are benefitting from staying on the treatment plan.

“*So there are those things [side effects] that I would consider changing if there was something that helped me to not have so many crises as I’m experiencing on the hydroxyurea. If there is something else that is better than the hydroxyurea that doesn’t have all the side effects, then yes, by all means*”

*(313, Female, 43yrs old)*.

Other participants were skeptical whether HU was “working” or managing their SCD complications. Some believed HU caused more complications. These participants did not feel their healthcare provider addressed these concerns.

For the most part, the primary reason for continuing this treatment option was the belief that HU, despite being unsatisfactory, is one of very few treatment options available to manage their advanced disease complications. The Group 2 participants appeared to lack information that could inform them as to the appropriate mechanism of action for HU.

#### 3.4.2. Group 2: Participants who were currently not taking HU

Two sub-groups of Group 2 participants were currently not taking HU. Twenty-three of the participants have never tried HU. A smaller number of participants tried HU in the past. Both groups, however, were skeptical about the safety and promised efficacy of the drug. Six participants who tried HU, stopped because their perception of an increase in pain crises, or the perception that HU had ceased to be effective. Five participants made the decision to stop the treatment plan without consulting their HCP.

In general, for the participants in Group 2 who had tried HU, but stopped due to skepticism over safety and efficacy, they did not report that they might try HU again at some point in the future. That is, they did not believe new information on HU would change their decision-making.

Although 23 participants in Group 2 had never tried HU, they too expressed skepticism about the side effects and complications from long-term use.

“*It might give you a little bit of relief now, but what is going to happen down the road? It’s kind of like, dealing with the evil you know versus the one you don’t know*”

*(320, Female, 32yrs)*.

Some participants who had never tried HU stated that they may try it sometime in the future. As with the participants currently taking HU, they were considering this treatment option because of the belief that HU is one of very few poor choices. Whether considering HU or not, some Group 2 participants were worried about HU since it was a chemotherapy drug and that it may cause hair loss and nausea.

“*I remembered that hydroxyurea is for people with cancer and I don’t have cancer so that’s why I’m saying no I don’t want that*.”

(382, Female, 48yr)

Group 2 participants not taking HU were also concerned that there would be side effects or “organ damage” from long-term use of HU. They were skeptical that all is known about long-term use of HU. They believed that either the physicians are not divulging this information or that research has not yet discovered long-term effects of taking HU.

Some participants believed that the use of HU in SCD was still experimental.

“*Now this may sound a bit morbid, sometimes we’ve got to sacrifice something, maybe even our self, for long-term benefits, not necessarily for our self but other people who are coming after us. Because I’m sure that every medication which we’re taking now, there were sets of people before us who took these medications. They were involved in trials and all of that and some that didn’t make it through the trial process, but here we are using the medications today. So, that’s my whole take on it*”

*(315, Male, 48yrs)*.

Group 2 participants discussed reluctance to start HU because of an aversion to taking pills or more medications. Much of the discussion for this group centered on self-managing their SCD or seeking homeopathic remedies. They were reluctant to start new drug therapies. Although not specially asked about their views on taking medication, across both groups, 17% (n = 16) of the participants explicitly discussed their aversion to taking medications. Of this response, 75% (n = 12) were the Group 2 participants. Furthermore, 35% (n = 33) discussed the alternative approaches that they are currently using, or their desire to learn more on this topic. Of these participants, 75% (n = 26) were adults in Group 2.

#### 3.4.3. Group 2: Information content- conversations with the physician

As with the Group 1 participants, Group 2 participants were asked the topics or information about SCD they would like to learn about. The majority of these participants expressed frustration with the lack of therapeutic options and cures. They researched information on new treatments and cures on their own, by searching the internet, attending conferences, and support groups. While many participants reported positive discussions with their physicians, they believed that they shouldered the responsibility for researching SCD. Some participants stated they believed healthcare providers lacked up-to-date information on treatments, such as the reduced intensity bone marrow transplant. Group 2 participants were concerned that physicians did not always recommend or discuss all available treatment options with them.

“*They [physicians] think that pain medication is the cure all for everything. That’s pretty much how to fix the medication, the pain medication when I’m having issues. They don’t really go over other medical options that I can take like when I hear about the hydroxyurea or the transplant information, they don’t really talk about that with me or new information that they find can help my type of sickle cell*”

*(20254, Female, 45yrs)*.

Because many Group 2 participants expressed an aversion to taking pills and new drug therapies, many indicated that they would like to learn alternative methods to addressing their symptoms focusing on a balanced diet, exercise, and specific foods, such as beets and carrots to alleviate symptoms. Participants in Group 2 were more likely than participants in Group 1 to be currently utilizing or actively seeking alternative approaches to managing their SCD complications. Aversion to taking pills also included aversion to taking pain medications.

## 4. Discussion

To the best of our knowledge, this is the first qualitative study of the relationship of patient age to the attitudes towards HU for SCD. We discovered distinct differences across the two age groups in the ways patients consider, discuss, or adopt HU. Group 2 adults were less likely to be currently taking HU. Group 2 adults who were currently taking or had tried HU in the past, were also more likely than Group 1 adults to report skepticism with efficacy and dissatisfaction with the side effects. Group 1 adults were more likely to be taking HU and were also less likely to report that they experienced any significant bad side effects. If they did experience any side effects, these were not a central to their attitude toward HU or central to their decision to start HU. Group 1 participants were also more likely to discuss the purpose and benefits as pivotal in the decision-making process and appeared to have a better understanding of its mechanism of action. They were more trusting that the benefits of staying on HU were measured over time by prevention or lessening of symptoms, such as pain and hospital stays. In contrast, the Group 2 participants were not as well informed about HU and reported concern about taking a chemotherapy drug. Group 2 adults currently taking HU were more likely to be skeptical about whether they were receiving any benefits from staying on the treatment. Group 2 adults were also less trusting that all of the side effects from HU are known or that healthcare providers divulged them. While the difference may in part represent a possible “era effect” in the practice of HU in SCD, this study was not designed to interrogate such an effect. The lack of utilization of HU may reflect Group 2 participants’ lack of awareness and lack of access to high quality medical care [15]. While more SCD patients are surviving to adulthood, patients often face challenges finding adult hematologists specializing in SCD [15]. For older adults, the aversion to taking medication may also reflect “treatment fatigue” from chronic care especially when it appears to be ineffective in ameliorating disease complications. Physical fatigue from treatment, such as chemotherapy, is well described, less is known, however, about “psychological fatigue associated with treatment engagement” [16]. The concept of treatment fatigue is best understood by the impact of various factors [17]. At the systems level, treatment fatigue may derive from lack of financial support or bureaucratic barriers in the healthcare system. At the physician level, poor physician-patient communication, mistrust, and barriers to receiving adequate treatment in emergency care all may contribute to treatment fatigue. At the patient level, treatment fatigue may be the result of “pill fatigue,” perception of lack of efficacy from medications, or impaired quality of life as a result of advanced SCD complications. Patient’s aversion to taking daily medications and wanting to take a break from the regimen is commonly understood as pill fatigue [18]. Patients may also develop pill fatigue and may stop or become irregular with taking medication because of the side effects, and the stress and burden of chronic medication administration.

Treatment fatigue has been studied in patients with HIV and in diabetes [16, 19, 20]. Patients describe treatment fatigue as “feeling overwhelmed by the cumulative effort of disease management”[16]. Treatment fatigue, or “burnout,” results in the increased burden of disease management [20]. In the case of HIV, the ongoing burden of maintaining antiretroviral therapy may result in “treatment regimen fatigue” [17]. The burden of daily self-management, as is the case with chronic diseases such as diabetes, may also lead to treatment fatigue [20, 21]. The concept of treatment fatigue has not previously been studied in the context of adults with SCD complications and may provide some insight into why Group 2 patients are skeptical to start HU or decide to stop the treatment plan.

Treatment fatigue may, but not always, result in treatment non-adherence which includes stopping the treatment plan or refusing to start treatment [22] [19]. Although adolescents have higher rates of non-adherence to treatment, in general, age is not strong predictor of treatment adherence [22, 23]. Many established underlying factors of treatment adherence, however, are applicable to older adults with SCD. As mentioned, adults with SCD are more likely to lack access to quality healthcare and may encounter stigmatization with they do [24, 25]. The physician-patient relationship is shown to have moderate impact on treatment adherence. When the physician-patient interaction is poor or lacking in pertinent information, patients are less likely to start or maintain the recommended treatment [22, 23]. Another factor influencing non-adherence to treatment is that patients may develop a set of beliefs and theories about their health and illness [23]. Many of the Group 2 participants in our study developed strategies for self-managing their disease complications. Unfortunately, self-management care may also work against some patients by causing less acceptance to the introduction of new treatments [23].

The skepticism of Group 2 adults towards HU may also be the result, at least in part, of a perceived lack of efficacy of HU in this age group. Development of chronic pain is related to age[26] with approximately thirty percent of adults with SCD having chronic pain[27]. HU alone may not ameliorate manifestation of the multi-organ damage which is more common in older adults[28]. These data provide a rationale for research examining the efficacy of HU in older adults.

At the other end of the spectrum, many of the Group 1 participants started HU as children with the parent(s) or legal guardian making the decision. Those who made the decision to start as young adults, offered positive accounts in regards to the decision to start HU and maintain the treatment. The Group 1 participants’ trust in the physician and staff may be a result of the comprehensive nature of pediatric care. Pediatric care resulted in monitoring the child’s health as well as educating the parent on complications of SCD and treatments such as HU and bone marrow transplant. Parent education and counseling has had a positive effect on morbidity and mortality [29, 30]. The data suggest that starting HU in childhood may be more likely to lead to long-term benefit and adherence.

A strength of our study was the in-depth analysis of adult patients’ attitudes towards HU in an era where there is increasing acceptance of HU in pediatric populations[31]. We believe that the data from this study will help tailor healthcare provider approaches to HU amongst SCD patients as they grow older. A limitation of this study is that participants were primarily recruited from national conferences. This group of SCD patients are often more engaged and motivated and may have more time and resources available than those with SCD who do not attend national conferences. The majority of this population reported they had a designated physician which may not be the case for the larger SCD population. Despite this access to resources and physicians, Group 2 participants, as compared to Group 1, were nonetheless less likely to be well-informed about HU and express skepticism about safety and efficacy of HU. Lastly, the sample was predominantly female, therefore they may not reflect the views of male adult patients with SCD.

## 5. Conclusion

Adults of different age groups with SCD have distinct attitudes towards HU, with younger adults more likely to report taking HU and reporting satisfaction and confidence regarding HU. The older adults, were less likely to report taking HU and more likely to be skeptical about its efficacy and concerned about benefits. The findings provide a rationale for further research to understand both efficacy of and acceptance of HU in older adults with SCD.
